# Efficacy comparison of concurrent chemoradiotherapy alone versus concurrent chemoradiotherapy plus adjuvant chemotherapy in stage III Locally Advanced Cervical Cancer: A single-center, retrospective observational cohort study

**DOI:** 10.1371/journal.pone.0354207

**Published:** 2026-08-10

**Authors:** Haizhou Yang, Yaxian Tan, Yajun Pang, Xiaowen Chen, Guoqiang Chen, Caixia Liang, Manyu Chen, Qiulong Liu, Zhihao Li, Jierong Xie, Zhennan Wang, Sihai Liao, Yufang Zuo

**Affiliations:** 1 Department of Gynecologic and Genitourinary Oncology, Affiliated Hospital of Guangdong Medical University, Zhanjiang, Guangdong Province, China; 2 Department of Oncology, Zizhong County Traditional Chinese Medicine Hospital, Neijiang, Sichuan Province, China; Indiana University School of Medicine, UNITED STATES OF AMERICA

## Abstract

**Objective:**

This study evaluated efficacy and safety of concurrent chemoradiotherapy (CCRT) versus CCRT plus adjuvant chemotherapy (CCRT+ACT) in 2018 Federation International of Gynecology and Obstetrics (FIGO) Stage III locally advanced cervical cancer (LACC) patients. This study provides clinical evidence to optimize treatment strategies for stage III LACC.

**Methods:**

A total of 72 patients diagnosed with stage III LACC were retrospectively enrolled from the Cancer Hospital of Guangdong Medical University between May 2018 and May 2024. They were divided into the CCRT group (n = 33) and the CCRT+ACT group (n = 39). Survival endpoints were analyzed using the Kaplan–Meier method with log-rank tests, and Cox proportional hazards regression models were applied to identify prognostic factors.

**Results:**

The 3-year OS (79.5% vs. 63.6%,*P* = 0.238) and PFS (56.4% vs. 45.5%,*P* = 0.706) rates showed no significant differences between the CCRT+ACT and CCRT groups. Kaplan–Meier survival analysis suggested that a positive lymph node size ≥ 10 mm (relative to a < 10 mm reference) may be associated with an increased hazard for mortality (HR = 8.235; *P* = 0.020) and disease progression (HR = 2.986; *P* = 0.020). Furthermore, abnormal post-brachytherapy SCC-Ag levels (> 1.5 ng/mL vs. ≤ 1.5 ng/mL reference) appeared to be strongly linked to adverse OS (HR = 13.641; *P* < 0.001) and PFS (HR = 4.722; *P* = 0.007). Regarding safety, CCRT+ACT did not increase grade 3–4 toxicities, suggesting a manageable safety profile.

**Conclusion:**

While CCRT+ACT did not significantly improve survival in the un-selected stage III LACC population, a positive lymph node size of ≥ 10 mm and elevated post-brachytherapy SCC-Ag levels may serve as important prognostic indicators for adverse outcomes. These findings are hypothesis-generating, suggesting that integrating these factors into clinical risk assessment might aid in refining the identification of high-risk subsets who could potentially benefit from more personalized, intensified adjuvant strategies in future prospective trials.

## Introduction

Cervical cancer remains a significant global health burden among women. 2022 global statistics reveal 660,000 newly diagnosed cases and 350,000 fatalities, accounting for a significant portion of female cancer mortality and ranking as the fourth most common female cancer [1]. As defined by the National Comprehensive Cancer Network (NCCN) guidelines [2], locally advanced cervical cancer (LACC) includes tumors staged IB3–IVA under the 2018 Federation International of Gynecology and Obstetrics (FIGO) criteria. Clinically, a narrower LACC subset (≥4 cm, cervix or upper vagina-confined, no metastasis) aligns with 2018 FIGO IB3 and IIA2 stages.

1999 landmark multicenter trials [3–7] established concurrent platinum-based chemoradiotherapy (CCRT) as the cornerstone of LACC management, showing a 30%−50% relative mortality reduction via synergistic effects. These findings led the NCCN to adopt CCRT as LACC’s standardized treatment [8], still in use today. Despite this, 30%−40% of LACC patients face post-treatment recurrence or metastasis risk [7], linked to poor tumor biology (e.g., low differentiation, lymph node metastasis, chemotherapy or radiotherapy resistance) [9]. To improve survival, researchers have explored intensified strategies, including neoadjuvant chemotherapy [10, 11] and adjuvant chemotherapy (ACT) [12, 13].

ACT efficacy in LACC remains debated. The OUTBACK trial [12] initially found no survival benefit, but post-hoc staging adjustments [14] revealed subgroup gains, suggesting earlier stage II-enriched studies may have masked ACT benefits [15–17]. Subsequent studies [14,18] reported survival improvements with ACT in lymph node-positive patients. A meta-analysis [19] demonstrated significant survival advantages with CCRT+ACT, while A 2018 FIGO staging-based model [20] suggested stage IVA LACC patients might derive greater benefit from CCRT+ACT.

To address this controversy, unlike prior studies, this research focuses on high-risk stage III LACC patients. We included 72 such patients treated at our center from May 2018 to May 2024, comparing CCRT vs CCRT+ACT outcomes and toxicities to assess intensified treatment value in this subgroup.

## Methods

### Study participants

**Fig 1:**
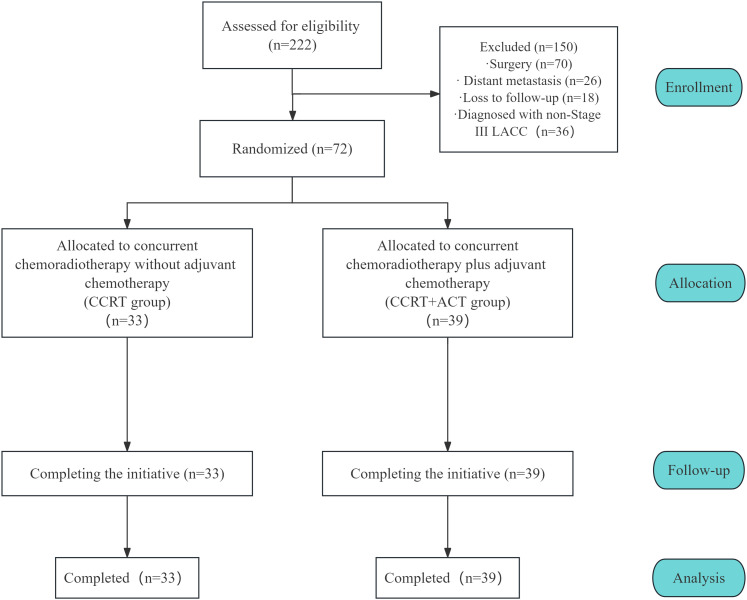
Study flow diagram. LACC: Locally Advanced Cervical Cancer.

This was a single-center, retrospective observational cohort study. Between May 2018 and May 2024, a total of 222 patients initially diagnosed with 2018 FIGO stage III LACC were identified at the Cancer Hospital of Guangdong Medical University. After applying our inclusion and exclusion criteria, 150 patients were excluded from the study. The primary reasons for exclusion were: 70 patients underwent surgery, 36 patients were diagnosed with non-stage III LACC, 26 patients had distant metastasis, and 18 patients were lost to follow-up..The final analyzed cohort consisted of 72 patients. Treatment assignment to either the CCRT group (n = 33) or the CCRT+ACT group (n = 39) was not randomized; it was determined based on physician discretion, grouping per inclusion and exclusion criteria, and institutional clinical guidelines at the time.Inclusion criteria were as follows: (1) Cervical cancer with histopathological confirmation of squamous cell or adenocarcinoma subtype, as determined by accredited pathology laboratories;(2) Aged between 38 and 77 years, with an Eastern Cooperative Oncology Group (ECOG) performance status of ≤2;(3) Staged IIIA-IIIC 2018 FIGO criteria via gynecological exam and imaging (e.g., MRI/CT) by two gynecologists;(4) Completed CCRT (with/without subsequent ACT) and signed pre-treatment consent. Exclusion criteria: (1) CCRT intolerance;(2) Pre-existing distant metastasis;(3) Incomplete data/loss to follow-up before treatment completion;(4) Prior surgery. The technical roadmap flowchart of the research is shown in Fig 1. Following the journal’s guidelines, we commit to making our data accessible to a team designated by the Editorial Board for supplementary analysis or reproducibility verification at other institutions, if requested.

### Treatment

CCRT Group: All patients received a standardized radiotherapy regimen comprising external beam radiotherapy (EBRT) and 3D high-dose-rate (HDR) brachytherapy. EBRT was delivered via volumetric modulated arc therapy (VMAT) using 6 MV photons, to a total dose of 45–50 Gy administered in 1.8–2.0 Gy fractions (five fractions per week). Target volumes and dose constraints were delineated according to RTOG guidelines [21]. The clinical target volume (CTV) encompassed the gross tumor, cervix, uterus, parametria, vagina, and regional lymph nodes. For patients with involved pelvic and/or para-aortic lymph nodes, an additional 10–15 Gy conformal boost was delivered to the positive nodes.

During EBRT, patients received concurrent systemic therapy. The standard regimen consisted of weekly intravenous cisplatin (30–40 mg/m^2^ for 1–5 cycles). For patients intolerant to cisplatin, alternative platinum agents (carboplatin or nedaplatin) were substituted. Systemic regimens broadly included: (1) single-agent cisplatin, or (2) a combination of liposomal/albumin-bound paclitaxel plus a platinum agent (cisplatin, carboplatin, or nedaplatin). Dose reductions or cycle delays were mandated in the event of grade 3–4 hematologic or gastrointestinal toxicities.

Following the completion of EBRT, 3D HDR intracavitary brachytherapy was performed using an Iridium-192 source via a Vienna applicator. Although historical Point A prescriptions (6–7 Gy/fraction for 4–5 fractions) were used as a reference, treatment planning was primarily optimized based on volumetric parameters. The median equivalent dose in 2 Gy fractions (EQD2) to the high-risk clinical target volume (HR-CTV D90) was 85 Gy. Radiation doses to organs at risk (OARs) were rigorously constrained, with the median D2cc limited to 80–90Gy for the bladder and 65–75 Gy for the rectum.

### Clinical efficacy and toxicity assessment

Post-treatment follow-ups were conducted on a quarterly to semi-annual basis via outpatient visits or telephone consultations. Routine monitoring for disease recurrence and metastasis included clinical examinations, hematological tests, pelvic magnetic resonance imaging (MRI), and chest/abdominal/pelvic computed tomography (CT). The efficacy evaluation period concluded in September 2025. Tumor response was formally assessed at 1-month post-treatment using the RECIST 1.1 criteria [22], categorizing outcomes as complete response (CR), partial response (PR), stable disease (SD), or progressive disease (PD). We deliberately selected this early 1-month assessment window—rather than the traditional 3-month timeline—to facilitate the prompt identification of non-responders for timely salvage interventions. However, we acknowledge that this accelerated timeline may capture ongoing radiation-induced acute inflammation and tumor necrosis, which could potentially underestimate the ultimate CR rate compared with later evaluations.

Treatment-related toxicities were rigorously monitored and documented. Systemic adverse events, including myelosuppression, gastrointestinal reactions, proteinuria, and transaminase elevation, were graded from I to IV according to the CTCAE 5.0 criteria [23]. Additionally, acute radiation-induced toxicities were evaluated using the RTOG/EORTC criteria [21].

### Statistical analysis

Statistical analysis employed SPSS 26.0. Categorical variables underwent chi-square or Fisher’s exact tests. Survival endpoints were analyzed via Kaplan-Meier curves with log-rank tests. Univariate and multivariate Cox regression identified prognostic factors, with *P* < 0.05 denoting significance.

### Ethics approval

This study was performed in line with the principles of the Declaration of Helsinki. Approval was granted by the Ethics Committee of the Affiliated Hospital of Guangdong Medical University (Date May 6th, 2025/No. PJKT2025−101).

## Results

This study enrolled 222 treatment-naive patients with 2018 FIGO stage III LACC from Cancer Hospital of Guangdong Medical University from May 2018 to May 2024, all completing CCRT or CCRT plus ACT. After screening, 72 patients were analyzed, divided into CCRT (n = 33) and CCRT+ACT (n = 39) groups. We observed no significant differences in age, FIGO stage, pathological type, lymph node metastasis, positive lymph node size, tumor size, post-brachytherapy response, or admission levels of SCC-Ag and CEA between the two groups.Among the 55 patients with lymph node metastasis, 45 patients had pelvic lymph node involvement only, while 10 patients had both pelvic and para-aortic lymph node involvement. Notably, 11 patients presented with clinically early-stage primary tumors (≤4 cm) but were upstaged to FIGO IIIC strictly due to positive lymph node disease. These findings confirm that the cohorts were well-balanced at baseline (see Table 1).

**Table 1:** Baseline characteristic comparison of the two patient groups.

Characteristics	All group	CCRT(*n* = 33)	CCRT+ACT(*n* = 39)	*χ²*	*P* value
Age(years)				0.914	0.339
≤ 65	54 (75%)	23 (69.7%)	31 (79.5%)		
> 65	18 (25%)	10 (30.3%)	8 (20.5%)		
FIGO Stage				1.331	0.722
IIIA	5 (6.9%)	2 (6.1%)	3 (7.7%)		
IIIB	12 (16.7%)	6 (18.2%)	6 (15.4%)		
IIIC1r	45 (62.5%)	22 (66.7%)	23 (59%)		
IIIC2r	10 (13.9%)	3 (9.1%)	7 (17.9%)		
Pathological type				0.906	0.341
Squamous cell carcinoma	60 (83.3%)	29（87.9%）	31 (79.5%)		
Adenocarcinoma	12 (16.7%)	4（12.1%）	8 (20.5%)		
Lymph node metastasis				1.510	0.219
No	17 (23.6%)	10 (30.3%)	7 (17.9%)		
Yes	55 (76.4%)	23 (69.7%)	32 (82.1%)		
Positive lymph node size(mm)				0.640	0.425
< 10	30（54.5%）	14（60.9%）	16（50%）		
≥ 10	25（45.5%）	9（39.1%）	16（50%）		
Tumor size（cm）				0.260	0.610
≤ 4	15 (20.8%)	6 (18.2%)	9 (25%)		
> 4	57 (79.2%)	27 (81.8%)	30 (75%)		
post-brachytherapy response				0.380	0.538
CR	20 (27.8%)	8 (24.2%)	12 (30.8%)		
PR	52 (72.2%)	25 (75.8%)	27 (69.2%)		
initial admission SCC-Ag(ng/mL)				0.690	0.405
≤ 1.5	9 (15%)	6 (20.7%)	3 (9.7%)		
> 1.5	51 (85%)	23 (79.3%)	28 (90.3%)		
post-brachytherapy SCC-Ag（ng/mL）				2.630	0.105
≤ 1.5	56 (93.3%)	25 (86.2%)	31 (100%)		
> 1.5	4 (6.7%)	4 (13.8%)	0 (0)		
initial admission CEA(ng/mL)				1.016	0.313
≤ 5	40 (69%)	19 (76%)	21 (63.6%)		
> 5	18 (31%)	6 (24%)	12 (36.4%)		

CCRT: Concurrent chemoradiotherapy; ACT: Adjuvant chemotherapy; CEA: Carcinoembryonic antigen; SCC-Ag: Squamous cell carcinoma antigen; FIGO: Federation International of Gynecology and Obstetrics; CR: Complete response; PR: Partial response.The data of SCC – Ag were exclusively obtained from patients with squamous cell carcinoma.

**Fig 2:**
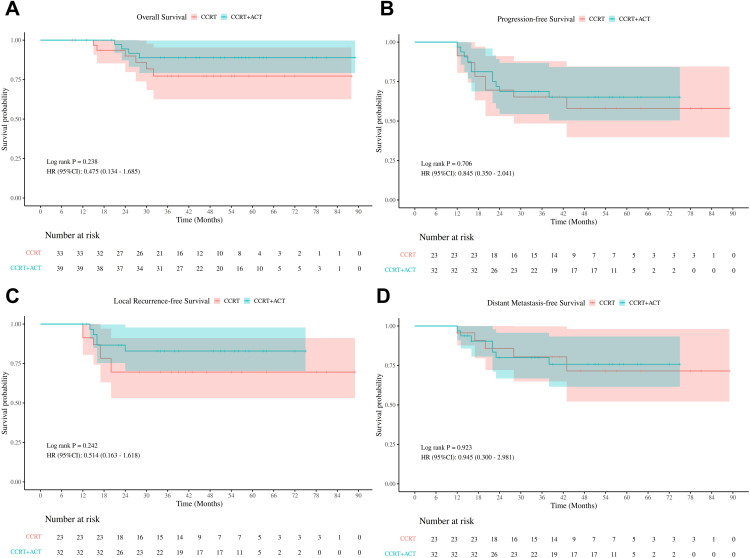
Comparison of survival curves between CCRT and CCRT+ACT groups. CCRT: Concurrent chemoradiotherapy; ACT: Adjuvant chemotherapy.

After a median follow-up duration of 41 months (range, 9–89 months), survival outcomes were compared between groups using Kaplan-Meier methods. For overall survival, the CCRT+ACT group demonstrated 1, 2, and 3 year rates of 100.0%, 94.9%, and 79.5%, respectively, compared with 100.0%, 81.8%, and 63.6% in the CCRT group (*P* = 0.238; Fig 2A). Regarding progression-free survival, the corresponding 1, 2, and 3 year rates were 82.1%, 66.7%, and 56.4% in the CCRT+ACT group versus 69.7%, 54.5%, and 45.5% in the CCRT group (*P* = 0.706; Fig 2B). For local recurrence-free survival, the 1 and 2 year rates were 82.1% and 66.7% in the CCRT+ACT group compared with 69.7% and 54.5% in the CCRT group (*P* = 0.242; Fig 2C). Similarly, the 1 and 2 year distant metastasis-free survival rates were 82.1% and 66.7% in the CCRT+ACT group versus 69.7% and 54.5% in the CCRT group (*P* = 0.923; Fig 2D). No statistically significant differences were observed across all survival endpoints.

**Fig 3:**
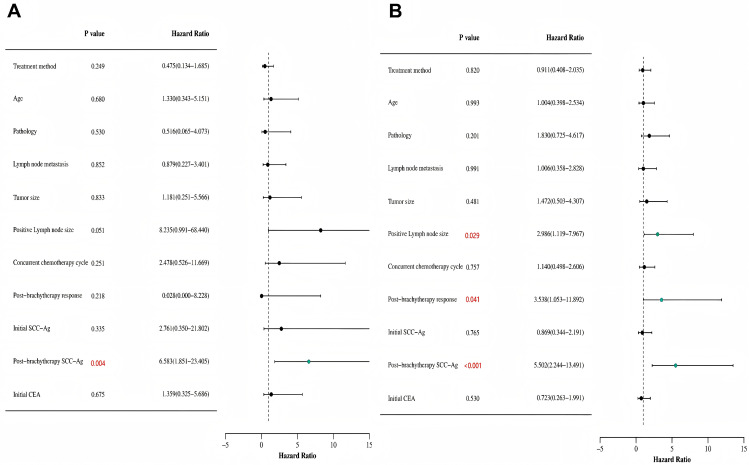
Forest plot of univariate analysis. A:Univariate analysis for overall survival;(B) Univariate analysis for progression-free survival; SCC-Ag: Squamous cell carcinoma antigen；CEA: Carcinoembryonic antigen.

Univariate analysis was conducted to identify prognostic factors associated with survival outcomes in patients with stage III LACC following CCRT. Variables assessed included: treatment modality, age, pathological type, lymph node metastasis status, primary tumor size, positive lymph node size, concurrent chemotherapy cycles, post-brachytherapy treatment response, baseline SCC-Ag level, post-brachytherapy SCC-Ag level, and baseline CEA level. Results revealed that post-brachytherapy SCC-Ag level was significantly associated with overall survival (*P* = 0.004, Fig 3A). Additionally, positive lymph node size, post-brachytherapy treatment response, and post-brachytherapy SCC-Ag level were significantly correlated with progression-free survival (*P* = 0.029, *P* = 0.041, and *P* < 0.001, respectively, Fig 3B).

To identify independent prognostic factors for survival outcomes in patients with LACC, a Cox proportional hazards regression model was constructed, incorporating clinically relevant baseline variables: treatment method, positive lymph node size, post-brachytherapy response, and post-brachytherapy SCC-Ag levels.In this model, positive lymph node size was identified as an independent risk factor for both overall survival and progression-free survival (*P* = 0.042;*P* = 0.008). Additionally, post-brachytherapy SCC-Ag levels independently predicted adverse overall survival and progression-free survival outcomes, with statistical significance (*P* = 0.009;*P* < 0.001).These findings indicate that positive lymph node size and post-brachytherapy SCC-Ag serve as robust independent predictors of survival in LACC patients, highlighting their potential clinical utility for risk stratification and treatment optimization.For details, refer to Table 2.

**Table 2:** Multivariate analysis affecting survival outcomes.

Factors	OS		PFS	
	*HR (95%CI)*	*P*	*HR (95%CI)*	*P*
Treatment method	2.207(0.239-20.347)	0.485	1.394(0.489-3.968）	0.534
Positive Lymph node size	14.453(1.102-189.637）	0.042*	4.272(1.468-12.434）	0.008*
post-brachytherapy SCC-Ag	19.151(2.122-172.835）	0.009*	11.096(3.151-39.072)	<0.001*
post-brachytherapy response	–	–	3.608(0.793-16.426）	0.097

OS: Overall Survival; PFS: Progression-Free Survival; SCC-Ag: Squamous cell carcinoma antigen.

**Fig 4:**
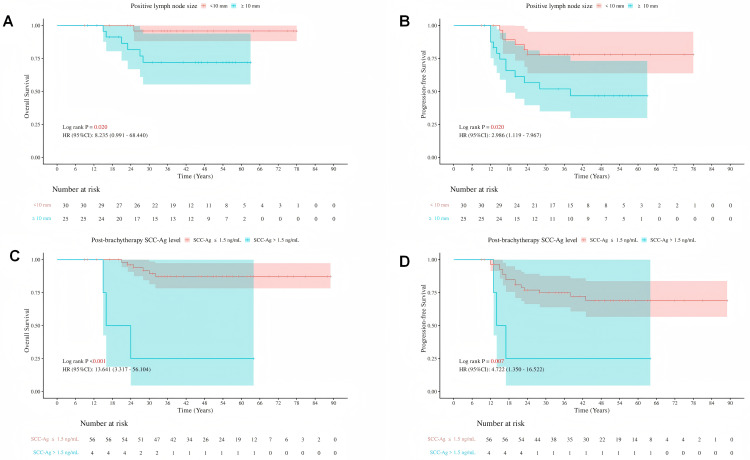
Prognostic Impact of Positive Lymph Node Size and Post-brachytherapy SCC-Ag Levels on Survival Outcomes. A Overall survival and B progression-free survival stratified by positive lymph node size (< 10 mm vs. ≥ 10 mm).C Overall survival and D progression-free survival stratified by post-brachytherapy SCC-Ag levels (≤ 1.5 ng/mL vs. > 1.5 ng/mL).

Kaplan–Meier survival analyses suggest that positive lymph node size and post-brachytherapy SCC-Ag levels are potential prognostic indicators in patients with stage III LACC. In these models, patients with a positive lymph node diameter < 10 mm were defined as the reference category. Compared to this reference, the group with nodes ≥ 10 mm appeared to have a higher risk of adverse outcomes, with estimated hazard ratios of 8.235 for overall survival (95% CI: 0.991–68.440, *P* = 0.020) (Fig 4A) and 2.986 for progression-free survival (95% CI: 1.119–7.967, *P* = 0.020) (Fig 4B). Similarly, using patients with post-brachytherapy SCC-Ag levels ≤ 1.5 ng/mL as the reference group, levels > 1.5 ng/mL were suggestive of significantly increased hazards for mortality (HR = 13.641, 95% CI: 3.317–56.104, *P* < 0.001) (Fig 4C)and disease progression (HR = 4.722, 95% CI: 1.350–16.522, *P* = 0.007) (Fig 4D). While these preliminary findings indicate that integrating nodal burden and dynamic biomarker monitoring might aid in risk stratification, they should be interpreted as hypothesis-generating given the retrospective nature of the study.

**Fig 5:**
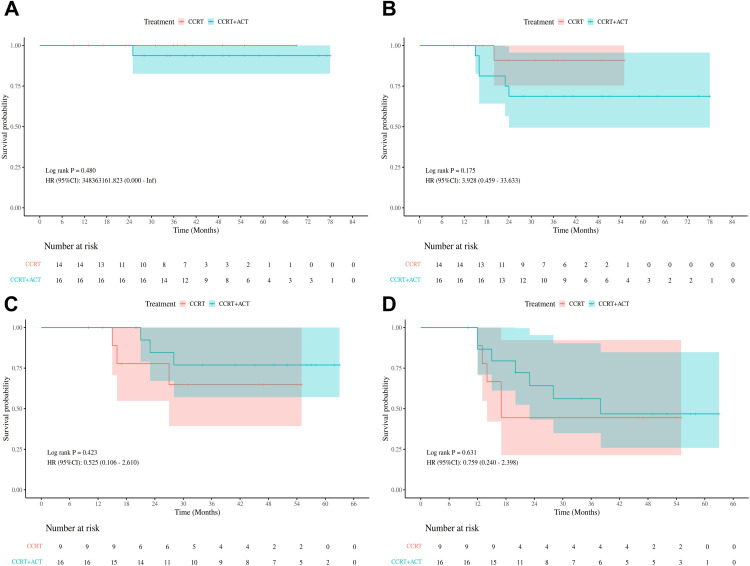
Survival Analysis of Treatment Modality in Lymph Node-Positive Patients with Different Lymph Node Sizes. A: Comparison of overall survival between CCRT and CCRT+ACT groups in <10 mm groups; B: Comparison of progression-free survival between CCRT and CCRT+ACT groups in <10 mm groups; C: Comparison of overall survival between CCRT and CCRT+ACT groups in ≥10 mm groups; D: Comparison of progression-free survival between CCRT and CCRT+ACT groups in ≥10 mm groups.

To evaluate the impact of treatment modalities (CCRT vs. CCRT+ACT) on survival outcomes in lymph node-positive patients stratified by lymph node size (≥10 mm vs. < 10 mm), subgroup analyses were performed. In the < 10 mm subgroup (Fig 5A, B), survival curves of the two treatment groups overlapped, with log-rank *P* values of 0.480 and 0.175, indicating no significant survival difference. Similarly, in the ≥ 10 mm subgroup (Fig 5C, D), no significant separation of survival curves was observed, with log-rank *P* values of 0.423 and 0.631; HR with 95% CI all included 1, confirming no statistically significant difference in survival benefits between regimens. Limitations include small subgroup sample sizes (e.g., 9 patients in the ≥ 10 mm CCRT group) leading to insufficient statistical power, and rapid late-stage patient attrition (e.g., 1 patient remaining in Panel A’s CCRT group at 72 months) affecting long-term analysis stability, highlighting the need for larger studies with extended follow-up for validation.

We compared late treatment-related toxicities between CCRT and CCRT+ACT, focusing on hematologic and non-hematologic adverse events (see Table 3). Toxicity rates did not differ significantly between groups (*P* > 0.05), indicating comparable safety profiles. Neutropenia was most common (CCRT vs. CCRT+ACT: 60.6% vs. 61.5% [grades 1–2]; 18.2% vs. 25.6% [grades 3–4], *χ²* = 1.544, *P* = 0.819), with leukopenia rates comparable (CCRT: 63.6%/33.3% [grades 1–2/3–4]; CCRT+ACT: 59%/35.9%, *χ²* = 1.616, *P* = 0.806). CCRT+ACT had slightly more grade 3–4 anemia (43.6% vs. 36.4%, *P* = 0.830) and CCRT more grade 3–4 thrombocytopenia (9.1% vs. 2.6%, *P* = 0.281); grade 1–2 AST elevation was marginally higher in CCRT+ACT (20.5% vs. 6.1%, *χ²* = 2.030, *P* = 0.154). Non-hematologic toxicities included proteinuria (CCRT: 42.5% vs. CCRT+ACT: 38.9%, *χ²* = 1.816, *P* = 0.611), more frequent grade 1–2 nausea/vomiting in CCRT+ACT (41.0% vs. 27.3%, *χ²* = 1.897, *P* = 0.387), and low comparable diarrhea rates (*P* = 0.802). Overall, CCRT+ACT did not increase grade 3–4 toxicities, suggesting manageable safety, though trends toward higher neutropenia and AST elevation in CCRT+ACT warrant attention.

**Table 3:** Toxic side effects of the two treatment groups.

	CCRT group *n* = 33 N(%)		CCRT+ACT group *n* = 39 N(%)		*χ²*	*P*
	1-2	3-4	1-2	3-4		
Hematologic toxicity						
Neutropenia	21 (60.6)	6 (18.2)	24 (61.5)	10 (25.6)	1.544	0.819
Hemoglobin reduction	17 (51.6)	12 (36.4)	15 (38.5)	17 (43.6)	1.483	0.830
Thrombocytopenia	12 (36.4)	3 (9.1)	10 (25.6)	1 (2.6)	5.061	0.281
AST	2 (6.1)	0 (0.0)	8 (20.5)	0 (0.0)	2.030	0.154
ALT	6 (18.2)	0 (0.0)	9 (23.1)	0 (0.0)	0.260	0.210
eGFR	15 (45.5)	0 (0.0)	20 (51.3)	0 (0.0)	0.243	0.886
Creatinine	6 (18.2)	0 (0.0)	7 (17.9)	1 (2.6)	2.124	0.547
Non-hematologic toxicity						
Proteinuria	14 (42.5)	1 (3.0)	28 (38.9)	0 (0.0)	1.816	0.611
Nausea and vomiting	9 (27.3)	1 (3.0)	16 (41.0)	2 (5.1)	1.897	0.387
Diarrhea	4 (12.1)	0 (0.0)	4 (10.3)	0 (0.0)	0.063	0.802

CCRT: Concurrent chemoradiotherapy; ACT: Adjuvant chemotherapy; AST: Aspartate Aminotransferase; eGFR: estimated Glomerular Filtration Rate; ALT: Alanine Aminotransferase.

## Discussion

### Results in the context of published literature

Our findings reflect the ongoing controversy regarding ACT in LACC. While Yavas et al. [24] and a meta-analysis of 1,659 patients [25] reported significant OS and PFS benefits with CCRT+ACT, the OUTBACK trial [12] found comparable 5-year OS between groups. However, post-hoc adjustments in OUTBACK suggested benefits in high-risk subsets, implying that including lower-risk stage II patients in earlier studies may have masked ACT efficacy [15–17]. Although our focus on stage III disease aligns with Fabri et al. [26] —who noted survival gains in lymph node-positive patients—we observed no significant benefit, likely due to our modest sample size (n = 72) and short follow-up (41 months).

The 27.8% complete response rate, lower than the literature-reported 54%–91% [27,28], is attributable to three factors.First, our 1-month efficacy assessment window is earlier than the traditional 3-month timeline; this may underestimate complete response as radiation-induced inflammation and necrosis often take longer to resolve.Second, 79.2% of our cohort presented with tumors >4 cm, which typically exhibit greater hypoxia and resistance, limiting drug penetration [29].Third, suboptimal delivery of the 5-cycle concurrent chemotherapy regimen may have weakened the synergistic radiosensitizing effect.

Unexpectedly, traditional factors like tumor size and lymph node metastasis did not reach statistical significance in this study. This may stem from a homogeneous cohort distribution reducing statistical power, or because nodal status was already incorporated into treatment stratification, thereby attenuating its independent prognostic impact.Furthermore, the anatomical focus of current FIGO staging may not fully capture the biological heterogeneity of these tumors, highlighting the need for more nuanced indicators like nodal size and dynamic biomarkers.

### Positive Lymph Node Size and Post-Brachytherapy SCC-Ag: Prognostic Factors and Clinical Significance in Stage III LACC

In our cohort, a positive lymph node size of ≥ 10 mm and post-brachytherapy SCC-Ag levels > 1.5 ng/mL were identified as potential prognostic factors for adverse outcomes. Using the < 10 mm group as the reference category, our findings suggest that a lymph node size of ≥ 10 mm may be associated with an increased hazard for mortality and disease progression, as indicated by corrected hazard ratios of 8.235 for overall survival and 2.986 for progression-free survival. This observation aligns with the premise that lymph node burden—represented here by the maximum short-axis diameter—may serve as an informative prognostic metric, consistent with prior studies linking larger nodal size to adverse survival [14]. Similarly, relative to the reference group (≤ 1.5 ng/mL), persistently elevated post-brachytherapy SCC-Ag (> 1.5 ng/mL) appeared to be associated with significantly higher risks of death and progression. This supports its potential role as a dynamic surrogate marker for incomplete tumor eradication or micrometastatic disease [17,30,31]. Regarding clinical application, these findings should be interpreted as hypothesis-generating. While CCRT+ACT did not significantly improve survival in the overall un-selected stage III population, the subset of patients presenting with multiple high-risk features (e.g., lymph nodes ≥ 10 mm and abnormal post-treatment SCC-Ag) might represent a group with an elevated risk of systemic failure. However, the interpretation of potential survival benefits from adjuvant therapies in this specific subgroup remains limited by our study’s small sample size (n = 72) and inherent treatment heterogeneity. Therefore, rather than directly guiding routine clinical practice, these parameters could serve as reference variables for risk stratification in future large-scale, prospective trials to further evaluate the efficacy of intensified systemic treatments in this population.

### Strengths and weaknesses

The study’s strengths stem from its targeted focus on stage III LACC, filling a key gap in the literature where prior investigations often included broader, more heterogeneous LACC stages [18,32,33]. By rigorously analyzing lymph node size and SCC-Ag as critical prognostic factors, our study offers actionable insights for personalized risk stratification. Additionally, comprehensive toxicity assessments validated the manageable safety profile of CCRT+ACT, confirming that there was no significant escalation in the prevalence of grade 3–4 treatment-emergent toxicities.

Despite these strengths, several limitations must be explicitly acknowledged. First, the retrospective, single-center design and the significant attrition rate during patient selection (from 222 initially identified to 72 analyzed) may introduce considerable selection bias. Second, the CCRT+ACT cohort exhibited notable treatment heterogeneity, including variable adjuvant chemotherapy regimens (single-agent vs. paclitaxel-based combinations), differing numbers of cycles, and concurrent platinum substitutions. This variability inherently weakens the causal interpretation of the survival outcomes and the null findings. Third, as a retrospective observational study, no a priori power and sample-size calculations were performed. The small sample size of 72 patients severely limits the statistical power of the study; consequently, our non-significant survival differences and subgroup analyses (e.g., lymph node burden stratification) are under-powered and must be interpreted with great caution. Fourth, the relatively short follow-up (median 36 months) may have underestimated long-term survival trends. Finally, relying on MRI for lymph node evaluation—which has a reported 40% micrometastasis miss rate [34] —instead of the more sensitive FDG PET-CT [35] risks overlooking high-risk patients, potentially biasing our risk stratification results.Furthermore, because our cohort underwent definitive chemoradiotherapy without surgical lymphadenectomy, we could not determine the exact absolute count of positive lymph nodes (due to the limitation of MRI in distinguishing conglomerated nodes), prompting our reliance on the maximum lymph node diameter (≥10 mm) and anatomical location as primary surrogate markers for nodal tumor burden.

### Implications for Practice and Future Research

Clinically, these studies reveal CCRT+ACT does not significantly improve survival in stage III LACC but shows a potential benefit trend, supporting lymph node size≥10 mm in lymph node-positive patients and post-brachytherapy SCC-Ag > 1.5 ng/mL as high-risk markers to guide intensified treatment. Future research should prioritize several key directions: First, multicenter, large-scale RCTs to validate ACT efficacy in high-risk subsets (e.g., ≥ 2 lymph nodes or size≥10 mm); second, FDG PET-CT for accurate lymph node assessment; third, molecular profiling (e.g., PD-L1, HPV) and immunotherapy (e.g., pembrolizumab [36], durvalumab [37]) to optimize personalized regimens; and finally quality-of-life evaluations to balance ACT benefits and toxicities.

## Conclusion

In conclusion, our study suggests that the addition of adjuvant chemotherapy following CCRT does not significantly improve OS or PFS in the un-selected population of stage III LACC patients. Nevertheless, a positive lymph node size of ≥ 10 mm and elevated post-brachytherapy SCC-Ag levels were identified as potent indicators of adverse outcomes compared to their respective reference categories (< 10 mm and ≤ 1.5 ng/mL). Specifically, these features appeared to be associated with significantly increased hazards for both mortality and disease progression. While these findings may aid in identifying high-risk subsets who could potentially benefit from intensified adjuvant strategies, they should be interpreted as hypothesis-generating due to the retrospective nature and limited sample size of this investigation. Further large-scale randomized controlled trials are warranted to refine treatment strategies and validate the efficacy of intensified care for these high-risk subgroups.

## References

[1] Bray F, Laversanne M, Sung H. Global cancer statistics 2022: GLOBOCAN estimates of incidence and mortality worldwide for 36 cancers in 185 countries. CA Cancer J Clin. 2024;74(3):229–63. doi: 10.3322/caac.21834 38572751

[2] National Comprehensive Cancer Network. NCCN clinical practice guidelines in oncology: cervical cancer (version 3. 2024). 2024. http://www.nccn.org

[3] Keys HM, Bundy BN, Stehman FB, Muderspach LI, Chafe WE, Suggs CL 3rd, et al. Cisplatin, radiation, and adjuvant hysterectomy compared with radiation and adjuvant hysterectomy for bulky stage IB cervical carcinoma. N Engl J Med. 1999;340(15):1154–61. doi: 10.1056/NEJM199904153401503 10202166

[4] Morris M, Eifel PJ, Lu J, Grigsby PW, Levenback C, Stevens RE, et al. Pelvic radiation with concurrent chemotherapy compared with pelvic and para-aortic radiation for high-risk cervical cancer. N Engl J Med. 1999;340(15):1137–43. doi: 10.1056/NEJM199904153401501 10202164

[5] Peters WA 3rd, Liu PY, Barrett RJ 2nd, Stock RJ, Monk BJ, Berek JS, et al. Concurrent chemotherapy and pelvic radiation therapy compared with pelvic radiation therapy alone as adjuvant therapy after radical surgery in high-risk early-stage cancer of the cervix. J Clin Oncol. 2000;18(8):1606–13. doi: 10.1200/JCO.2000.18.8.1606 10764420

[6] Rose PG, Bundy BN, Watkins EB, Thigpen JT, Deppe G, Maiman MA, et al. Concurrent cisplatin-based radiotherapy and chemotherapy for locally advanced cervical cancer. N Engl J Med. 1999;340(15):1144–53. doi: 10.1056/NEJM199904153401502 10202165

[7] Whitney CW, Sause W, Bundy BN, Malfetano JH, Hannigan EV, Fowler WC Jr, et al. Randomized comparison of fluorouracil plus cisplatin versus hydroxyurea as an adjunct to radiation therapy in stage IIB-IVA carcinoma of the cervix with negative para-aortic lymph nodes: a Gynecologic Oncology Group and Southwest Oncology Group study. J Clin Oncol. 1999;17(5):1339–48. doi: 10.1200/JCO.1999.17.5.1339 10334517

[8] Koh WJ, Abu-Rustum NR, Bean S, et al. Cervical cancer, version 3.2019, NCCN clinical practice guidelines in oncology. J Natl Compr Canc Netw. 2019;17(1):64–84. doi: 10.6004/jnccn.2019.0001 30659131

[9] Tu K, Chen C, Cheng X, et al. Comparison of the curative effect and safety of consolidation chemotherapy after concurrent chemoradiotherapy with concurrent chemoradiotherapy alone for locally advanced cervical cancer. Eur J Gynaecol Oncol. 2018;39(4):2018.

[10] Kenter GG, Greggi S, Vergote I, Katsaros D, Kobierski J, van Doorn H, et al. Randomized Phase III Study Comparing Neoadjuvant Chemotherapy Followed by Surgery Versus Chemoradiation in Stage IB2-IIB Cervical Cancer: EORTC-55994. J Clin Oncol. 2023;41(32):5035–43. doi: 10.1200/JCO.22.02852 37656948

[11] Gupta S, Maheshwari A, Parab P, Mahantshetty U, Hawaldar R, Sastri Chopra S, et al. Neoadjuvant chemotherapy followed by radical surgery versus concomitant chemotherapy and radiotherapy in patients with stage IB2, IIA, or IIB squamous cervical cancer: a randomized controlled trial. J Clin Oncol. 2018;36(16):1548–55. doi: 10.1200/JCO.2017.75.9985 29432076

[12] Mileshkin LR, Moore KN, Barnes EH, Gebski V, Narayan K, King MT, et al. Adjuvant chemotherapy following chemoradiotherapy as primary treatment for locally advanced cervical cancer versus chemoradiotherapy alone (OUTBACK): an international, open-label, randomised, phase 3 trial. Lancet Oncol. 2023;24(5):468–82. doi: 10.1016/S1470-2045(23)00147-X 37080223 PMC11075114

[13] Dueñas-González A, Zarbá JJ, Patel F, Alcedo JC, Beslija S, Casanova L, et al. Phase III, open-label, randomized study comparing concurrent gemcitabine plus cisplatin and radiation followed by adjuvant gemcitabine and cisplatin versus concurrent cisplatin and radiation in patients with stage IIB to IVA carcinoma of the cervix. J Clin Oncol. 2011;29(13):1678–85. doi: 10.1200/JCO.2009.25.9663 21444871

[14] Mileshkin LR, Moore KN, Barnes EH, Lee YC, Gebski V, Narayan K, et al. Staging locally advanced cervical cancer with FIGO 2018 versus FIGO 2008: Impact on overall survival and progression-free survival in the OUTBACK trial (ANZGOG 0902, RTOG 1174, NRG 0274). JCO. 2022;40(16_suppl):5531–5531. doi: 10.1200/jco.2022.40.16_suppl.5531

[15] Tovanabutra C, Asakij T, Rongsriyam K, Tangjitgamol S, Tharavichitkul E, Sukhaboon J, et al. Long-Term Outcomes and Sites of Failure in Locally Advanced, Cervical Cancer Patients Treated by Concurrent Chemoradiation with or without Adjuvant Chemotherapy: ACTLACC Trial. Asian Pac J Cancer Prev. 2021;22(9):2977–85. doi: 10.31557/APJCP.2021.22.9.2977 34582670 PMC8850888

[16] Kou L, Zhang T, Yang X, Peng S, Wang Y, Yuan M, et al. Role of adjuvant chemotherapy after concurrent chemoradiotherapy in patients with locally advanced cervical cancer. Future Oncol. 2022;18(16):1917–1915. doi: 10.2217/fon-2021-0818 35193379

[17] Wu N, Su X, Song H, Li Y, Gu F, Sun X, et al. A Multi-Institutional Retrospective Analysis of Oncologic Outcomes for Patients With Locally Advanced Cervical Cancer Undergoing Platinum-Based Adjuvant Chemotherapy After Concurrent Chemoradiotherapy. Cancer Control. 2021;28:1073274821989307. doi: 10.1177/1073274821989307 33593091 PMC8482744

[18] Wang Y-N, Zhong M-L, Liang M-R, Yang J-T, Zeng S-Y. The Therapeutic Value of Adjuvant Chemotherapy after Concurrent Chemoradiotherapy for Locally Advanced Cervical Cancer. Gynecol Obstet Invest. 2023;88(5):286–93. doi: 10.1159/000533122 37497957

[19] Ma X, Fang J, Zhang L, Huang Y, Shen H, Ma X, et al. Efficacy and safety of adjuvant chemotherapy for locally advanced cervical cancer: A systematic review and meta-analysis. Crit Rev Oncol Hematol. 2023;184:103953. doi: 10.1016/j.critrevonc.2023.103953 36889613

[20] Hua L, Wei M, Feng C, Li S, Wen X, Chen S. Nomogram for Predicting Survival in Locally Advanced Cervical Cancer with Concurrent Chemoradiotherapy plus or Not Adjuvant Chemotherapy: A Retrospective Analysis Based on 2018 FIGO Staging. Cancer Biother Radiopharm. 2024;39(9):690–705. doi: 10.1089/cbr.2023.0199 38828494

[21] Shaw E, Kline R, Gillin M. Radiation therapy oncology group: radiosurgery quality assurance guidelines. Int J Radiat Oncol Biol Phys. 1993;27(5):1231–9. doi: 10.1016/0360-3016(93)90548-a 8262852

[22] Eisenhauer EA, Therasse P, Bogaerts J, Schwartz LH, Sargent D, Ford R, et al. New response evaluation criteria in solid tumours: revised RECIST guideline (version 1.1). Eur J Cancer. 2009;45(2):228–47. doi: 10.1016/j.ejca.2008.10.026 19097774

[23] Freites-Martinez A, Santana N, Arias-Santiago S, Viera A. Using the Common Terminology Criteria for Adverse Events (CTCAE - Version 5.0) to Evaluate the Severity of Adverse Events of Anticancer Therapies. Actas Dermosifiliogr (Engl Ed). 2021;112(1):90–2. doi: 10.1016/j.ad.2019.05.009 32891586

[24] Yavas G, Yavas C, Sen E, Oner I, Celik C, Ata O. Adjuvant carboplatin and paclitaxel after concurrent cisplatin and radiotherapy in patients with locally advanced cervical cancer. Int J Gynecol Cancer. 2019;29(1):42–7. doi: 10.1136/ijgc-2018-000022 30640682

[25] Zhong L, Li K, Song L, Yin R. The effect of consolidation chemotherapy after concurrent chemoradiation on the prognosis of locally advanced cervical cancer: a systematic review and meta-analysis. J Obstet Gynaecol. 2022;42(5):830–7. doi: 10.1080/01443615.2021.2012437 35148230

[26] Fabri VA, Queiroz ACM, Mantoan H. The impact of addition of consolidation chemotherapy to standard cisplatin-based chemoradiotherapy in uterine cervical cancer: matter of distant relapse. J Oncol. 2019;2019:1217838. doi: 10.1155/2019/1217838 30984261 PMC6432701

[27] Datta NR, Stutz E, Liu M, Rogers S, Klingbiel D, Siebenhüner A, et al. Concurrent chemoradiotherapy vs. radiotherapy alone in locally advanced cervix cancer: A systematic review and meta-analysis. Gynecol Oncol. 2017;145(2):374–85. doi: 10.1016/j.ygyno.2017.01.033 28188016

[28] Tangjitgamol S, Katanyoo K, Laopaiboon M, Lumbiganon P, Manusirivithaya S, Supawattanabodee B. Adjuvant chemotherapy after concurrent chemoradiation for locally advanced cervical cancer. Cochrane Database Syst Rev. 2014;2014(12):CD010401. doi: 10.1002/14651858.CD010401.pub2 25470408 PMC6402532

[29] Hequet D, Marchand E, Place V, Fourchotte V, De La Rochefordière A, Dridi S, et al. Evaluation and impact of residual disease in locally advanced cervical cancer after concurrent chemoradiation therapy: results of a multicenter study. Eur J Surg Oncol. 2013;39(12):1428–34. doi: 10.1016/j.ejso.2013.10.006 24183796

[30] Fu J, Wang W, Wang Y, Liu C, Wang P. The role of squamous cell carcinoma antigen (SCC Ag) in outcome prediction after concurrent chemoradiotherapy and treatment decisions for patients with cervical cancer. Radiat Oncol. 2019;14(1):146. doi: 10.1186/s13014-019-1355-4 31416463 PMC6694518

[31] Tony V, Sathyamurthy A, Ramireddy JK, Iswarya SJ, Gowri SM, Thomas A, et al. Role of squamous cell carcinoma antigen in prognostication, monitoring of treatment response, and surveillance of locally advanced cervical carcinoma. J Cancer Res Ther. 2023;19(5):1236–40. doi: 10.4103/jcrt.jcrt_335_21 37787289

[32] McComas KN, Torgeson AM, Ager BJ, Hellekson C, Burt LM, Maurer KA, et al. The variable impact of positive lymph nodes in cervical cancer: Implications of the new FIGO staging system. Gynecol Oncol. 2020;156(1):85–92. doi: 10.1016/j.ygyno.2019.10.025 31744640

[33] Chemoradiotherapy for Cervical Cancer Meta-Analysis Collaboration. Reducing uncertainties about the effects of chemoradiotherapy for cervical cancer: a systematic review and meta-analysis of individual patient data from 18 randomized trials. J Clin Oncol. 2008;26(35):5802–12. doi: 10.1200/JCO.2008.16.4368 19001332 PMC2645100

[34] Benedetti Panici P, Basile S, Angioli R. Pelvic and aortic lymphadenectomy in cervical cancer: the standardization of surgical procedure and its clinical impact. Gynecol Oncol. 2009;113(2):284–90. doi: 10.1016/j.ygyno.2008.12.014 19157526

[35] Dag Z, Yilmaz B, Dogan AK, Aksan DU, Ozkurt H, Kızılkaya HO, et al. Comparison of the prognostic value of F-18 FDG PET/CT metabolic parameters of primary tumors and MRI findings in patients with locally advanced cervical cancer treated with concurrent chemoradiotherapy. Brachytherapy. 2019;18(2):154–62. doi: 10.1016/j.brachy.2018.11.005 30594422

[36] Lorusso D, Xiang Y, Hasegawa K, Scambia G, Leiva M, Ramos-Elias P, et al. Pembrolizumab or placebo with chemoradiotherapy followed by pembrolizumab or placebo for newly diagnosed, high-risk, locally advanced cervical cancer (ENGOT-cx11/GOG-3047/KEYNOTE-A18): a randomised, double-blind, phase 3 clinical trial. Lancet. 2024;403(10434):1341–50. doi: 10.1016/S0140-6736(24)00317-9 38521086

[37] Monk BJ, Toita T, Wu X, Vázquez Limón JC, Tarnawski R, Mandai M, et al. Durvalumab versus placebo with chemoradiotherapy for locally advanced cervical cancer (CALLA): a randomised, double-blind, phase 3 trial. Lancet Oncol. 2023;24(12):1334–48. doi: 10.1016/S1470-2045(23)00479-5 38039991

